# Deriving Optimal Treatment Timing for Adaptive Therapy: Matching the Model to the Tumor Dynamics

**DOI:** 10.1007/s11538-025-01525-y

**Published:** 2025-09-08

**Authors:** Kit Gallagher, Maximilian A. R. Strobl, Alexander R. A. Anderson, Philip K. Maini

**Affiliations:** 1 Wolfson Centre for Mathematical Biology, Mathematical Institute, Oxford, UK; 2 Integrated Mathematical Oncology, Moffitt Cancer Center, Florida, USA; 3 Theory Division, Cleveland Clinic, Ohio, USA

**Keywords:** Adaptive Therapy, Mathematical Oncology, Optimal Treatment Schedules, Lotka–Volterra, Competition Models, Time-Varying Threshold

## Abstract

Adaptive therapy (AT) protocols have been introduced to combat drug resistance in cancer, and are characterized by breaks from maximum tolerated dose treatment (the current standard of care in most clinical settings). These breaks are scheduled to maintain tolerably high levels of tumor burden, employing competitive suppression of treatment-resistant sub-populations by treatment-sensitive sub-populations. AT has been integrated into several ongoing or planned clinical trials, including treatment of metastatic castrate-resistant prostate cancer, ovarian cancer, and BRAF-mutant melanoma, with initial clinical results suggesting that it can offer significant extensions in the time to progression over the standard of care. Prior AT protocols apply drug treatment when the tumor is within a specific size window, typically determined by the initial tumor size. However, this approach may be sub-optimal as it does not account for variation in tumor dynamics between patients, resulting in significant heterogeneity in patient outcomes. Mathematical modeling and analysis have been proposed to optimize adaptive protocols, but they do not account for clinical restrictions, most notably the discrete time intervals between the clinical appointments where a patient’s tumor burden is measured and their treatment schedule is re-evaluated. We present a general framework for deriving optimal treatment protocols that account for these discrete time intervals, and derive optimal schedules for several models to avoid model-specific personalization. We identify a trade-off between the frequency of patient monitoring and the time to progression attainable, and propose an AT protocol that determines drug dosing based on a patient-specific threshold for tumor size. Finally, we identify a subset of patients with qualitatively different dynamics that instead require a novel AT protocol based on a threshold that changes over the course of treatment.

**Supplementary Information:**

The online version contains supplementary material available at 10.1007/s11538-025-01525-y.

## Introduction

Adaptive therapy (AT) is a new paradigm in cancer treatment that uses principles from evolution to delay disease progression for late-stage cancer patients. Cancerous tumors are highly genetically heterogeneous (Allison and Sledge 2014), comprised of many different clones with potentially differing resistance to any given therapeutic agent (Turner and Reis-Filho 2012). This heterogeneity enables tumors to be considered through the lens of Darwinian evolution, wherein multiple species with different (environment-dependent) fitness compete with each other (Gerlinger and Swanton 2010). In particular, competition (be it for resources, space to grow, or access to vasculature) between clones of variable drug resistance can reduce growth rates and result in ‘competitive suppression’ of all clones, including drug-resistant clones that are otherwise unencumbered by the application of treatment. Suppression may be further enhanced by naturally lower base proliferation rates of drug-resistant cells relative to sensitive cells (a ‘cost of resistance’), as observed *in vivo* across several tumor cell lines (Enriquez-Navas et al. 2016; Bacevic et al. 2017).

Treatment breaks/holidays may leverage this competitive suppression to gain indirect control over drug-resistant cells. While drug-sensitive clones can be controlled directly by the application of treatment, the growth of resistant clones can only be limited indirectly, via competitive suppression from the drug-sensitive cells. These treatment breaks allow regrowth of the drug-sensitive population, renewing the competitive suppression of resistant cells and re-sensitizing the tumor to treatment. In contrast to previous clinical implementations of intermittent therapy (Hussain et al. 2013; Crook et al. 2012), wherein routine drug holidays of a fixed length were scheduled uniformly across the whole patient cohort, AT tailors drug scheduling to an individual patient’s tumor dynamics (Gatenby et al. 2009). These adaptive strategies exploit both spatial and resource competition between drug-sensitive and -resistant cells (Gallaher et al. 2018; Bacevic et al. 2017; Strobl et al. 2022).

**Fig. 1:**
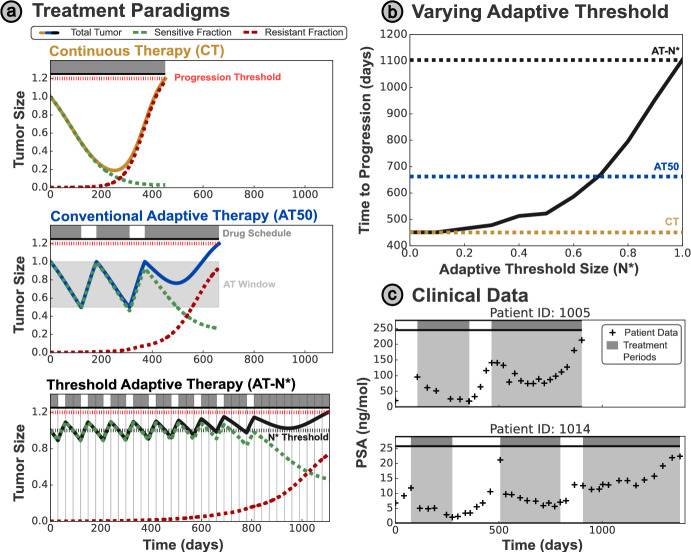
**(a)** Comparison of different treatment protocols, simulated using the Lotka–Volterra model as defined in Section 2.1.1. In each case, tumor progression is defined as $20\%$ growth from the initial size, and the tumor size is normalized relative to the initial tumor size. Continuous treatment is the clinical standard of care; however, progression can be delayed by introducing breaks in treatment. In conventional (‘window-based’) AT, a treatment holiday is initiated when the tumor has halved in size, and resumed when the tumor returns to the initial size. We propose a modified AT protocol (AT-N*) based on a single threshold $N^{*}$ - treatment is only delivered when the tumor is larger than this threshold. Overshooting of the threshold occurs as treatment may only be modified every 30 days (denoted by vertical gray lines). **(b)** Higher treatment thresholds in AT-N* correspond to greatly delayed progression, with significant improvement over the standard of care. **(c)** We illustrate the clinical application of AT, showing the outcomes on two exemplar patients from a clinical trial on metastatic prostate cancer based on the AT50 protocol (Zhang et al. 2022)

The advantage of AT over continuous treatment (CT) is exemplified in Figure 1a. We consider a conventional AT protocol, termed AT50, which applies treatment until the tumor is $50\%$ of the original size, before withdrawing treatment until the tumor regains its initial size. We will refer to this formulation of AT as ‘window-based’ AT, as the tumor size is maintained within a set window based on the initial tumor size, and a formal definition of this protocol is given in Section 2.2. The ‘window-based’ AT protocol has been applied clinically to metastatic, castrate-resistant prostate cancer, where a two-fold increase in the mean time to progression (TTP) was observed, broadly in agreement with the simulated benefit here (Zhang et al. 2022).

These benefits are achieved by maintaining a larger sensitive cell population to continually suppress the growth of resistant cells. This suppression has been discussed by previous analytic work, which shows that the TTP may be maximized by maintaining as large a sensitive sub-population as possible (Hansen et al. 2017; Viossat and Noble 2021), below some upper bound on the tumor size (given by the progression limit). Hansen and Read have even proposed setting the upper limit of ‘window-based’ AT above the initial size (Hansen and Read 2020), based on the proposal that patients may tolerate slight increases in the tumor burden above the initial size, and this has since been implemented as ‘Range-Bounded’ Adaptive Therapy (Brady-Nicholls and Enderling 2022).

However, these studies assume continuous monitoring of the tumor size, allowing the drug to be withdrawn/reapplied as soon as the tumor size crosses a pre-defined threshold. With the development of wearable devices, this may be a realistic assumption in the future; however, it is not currently possible in the clinic. We show in Section 2.3 that these optimal frameworks break down in clinically realistic settings, where updates to the treatment schedule can only be made at discrete time points, and the tumor size is not tracked between these time points. These limitations motivate revision of previous optimal AT protocols, which relied upon continuous monitoring and decision-making capability to maintain a maximal sensitive subpopulation.

We propose an alternate strategy to maintain a higher average tumor size than ‘window-based’ AT approaches: a ‘threshold-based’ strategy (AT-N*). In this approach, treatment is given only when the tumor exceeds a pre-specified threshold size ($N^{*}$). As shown in Figure 1a, using $N^{*}$ as the initial tumor size, AT-N* can significantly increase TTP compared to both CT and AT50. The benefit of AT-N* depends on the threshold size $N^{*}$ used; higher thresholds achieve greater TTP values by maintaining a larger sensitive cell population (Figure 1b).

The AT-N* protocol may be updated at discrete intervals (e.g., every $\tau = 30$ days, as shown in Figure 1a), reflecting limitations in the clinical implementation of mathematically-derived treatment protocols. The threshold $N^{*}$ can also be tailored to each patient, accounting for variation in tumor dynamics that has been observed in clinical AT trials (Zhang et al. 2022) and visualized in Figure 1c.

In this paper, we present a framework to derive an optimal treatment threshold based on the discrete time interval between treatment updates, before applying this framework to a range of mathematical tumor models with different underlying assumptions. In each model we consider, the cancer is incurable since there is a drug-resistant species that cannot be entirely eliminated, and the null state ($S = R = 0$) is unstable. This incurability is a common phenomenon in metastatic cancers with existing treatment resistance; instead of seeking to cure the cancer, we therefore aim to control its growth to increase the TTP. We focus on binary dosing schedules, where treatment is either given at the (clinically-approved) maximum tolerated dose or a treatment holiday is given. While approaches that modulate the prescribed dose are the subject of several ongoing clinical trials (Mukherjee et al. 2024; Gallaher et al. 2015), clinical data remain limited. Given the sensitivity of the optimal formulation to the dose response curve (West et al. 2020), which is not well-characterized in many cases, we only consider the well-established binary dosing schedules that have been validated in prior clinical trials (Bruchovsky et al. 2006; Zhang et al. 2017).

We apply our framework to the clinical context of metastatic prostate cancer. We chose this context in part because of a clear clinical need for strategies that extend TTP, given low survival rates; the 5-year survival rate of metastatic castration-resistant prostate cancer is approximately 30% (Huo et al. 2025). Furthermore, the availability of prostate-specific antigen (PSA) levels provides an accessible and widely accepted marker of tumor burden in this context (Adhyam and Gupta 2012; de Bono et al. 2011) and allows regular and non-invasive tracking of tumor size to inform the AT protocol. The mathematical tumor models we discuss below were all previously proposed to describe the application of adaptive therapy to metastatic prostate cancer, based on data from a Phase II trial of intermittent androgen suppression for locally-advanced prostate cancer (Bruchovsky et al. 2006). This treatment consists of twice-daily tablets of cyproterone acetate, which may be administered at home, allowing treatment to be maintained between clinical appointments. Nevertheless, to preserve clinical realism, we only allow a patient’s treatment to be changed (e.g., switched from treatment to holiday) at discrete intervals, tied to the clinical appointments where the tumor burden is measured from the PSA.

We find that accounting for discrete intervals between appointments requires highly patient-specific optimal thresholds, motivating the need for personalized approaches to adaptive therapy. This paper provides an analytic approach to evaluating patient-specific optimal treatment protocols based on the parameters of the tumor dynamics. We also show that the optimal treatment protocols depend on the underlying model assumptions – for example, a model that captures systematic changes in the patient’s tumor dynamics over time requires a time-varying optimal treatment threshold. Overall, we develop an approach to incorporate clinical realities into mathematical analysis of optimal treatment schedules, and present optimal treatment approaches for a range of competition-based tumor models.

## Methods

In this section, we introduce three distinct ordinary differential equation models for the response of a heterogeneous tumor to therapy. In each case, the heterogeneity in drug response is captured by two separate populations, which are subject to some form of inter-species competition. Each model was previously developed to capture the drug response dynamics of patients undergoing intermittent/adaptive scheduling of hormone therapy for metastatic prostate cancer.

### Tumor Models

#### Lotka–Volterra Model

Strobl et al. (2021) model heterogeneity in drug response within a partially drug-resistant tumor by two competing cell types: drug-sensitive cells *S*(*t*), and fully resistant cells *R*(*t*), via the following two population Lotka–Volterra model (Lotka 1910; Volterra 1928):

1 $$\begin{aligned} \frac{dS}{dt}&= r_{S} S \left( 1 - \frac{S+R}{K}\right) \times (1-d_{D}D) - dS, \nonumber \\ \frac{dR}{dt}&= r_{R} R \left( 1 - \frac{S+R}{K}\right) - dR. \end{aligned}$$

Competition is represented by a logistic growth model with a shared carrying capacity *K*, while each species has a separate growth rate ($r_{S}$ and $r_{R}$ respectively). Cells are assumed to die naturally at the same rate *d*, while the Norton–Simon model (Norton and Simon 1977) is adopted to model the drug-induced killing of sensitive cells, occurring at a rate proportional to the population’s growth rate and the drug concentration, *D*(*t*) (with proportionality factor $d_D$).

This model only considers pre-existing resistance, and there is no mechanism for the acquisition of drug resistance during treatment. However, the inclusion of genetic mutations in simple models has been shown to have no significant bearing on the overall response to therapy (Viossat and Noble 2021). Parameter values were adopted from Strobl et al. (2021) and are given in Table S1. Note that throughout this paper, we will use *N* to denote the total tumor size - i.e., $N = S + R$.

#### Waning Competition Model

In comparison, we also consider a modified Lotka–Volterra model proposed by Lu et al. (2024). This time-varied generalized Lotka–Volterra model (with growth scaling exponent $\alpha$) also describes separate drug-sensitive and drug-resistant cell populations competing for shared resources in the tumor microenvironment. However, this model considers a modified logistic growth term with exponentially decreasing resource overlap, described by the resistance index $\gamma > 0$ (henceforth this model will be referred to as the ‘Waning Competition’ model). Lu et al. (2024) attribute this trend to competition-induced mutations and epigenetic modifications within the cancer population, such that competition intensity weakens over time as there are fewer shared resources. Explicitly, this model may be written (in non-dimensional form) as:

2 $$\begin{aligned} \begin{gathered} \frac{dS}{dt} = r_{S} S \left[ 1 - \left( \frac{S + \frac{R}{1 + e^{\gamma t}}}{K_{S}}\right) ^{\alpha } - d_{S}D \right] , \\ \frac{dR}{dt} = r_{R} R \left[ 1 - \left( \frac{R + \frac{S}{1 + e^{\gamma t}}}{K_{R}}\right) ^{\alpha } - d_{R}D \right] , \end{gathered}\end{aligned}$$

where *S* and *R* are the sensitive and resistant cell sub-populations respectively, and *D* is the drug concentration. Each cell species *i* has a distinct growth rate $r_{i}$, carrying capacity $K_{i}$, and drug-induced death rate $d_{i}$. It is worth noting that this model also reduces to the generalized logistic model in the case where $\gamma =0$ (eliminating the explicitly time-dependent competition), and in the absence of treatment.

Lu et al. (2024) defined progression for this model as the growth of the resistant population to $R(t) \ge 0.1 K_{R}$. While we retain this definition, it is also necessary to set an upper limit $N(t) < 1.2N_{0}$ on the allowable total tumor size, as it would otherwise be optimal to allow arbitrarily large drug-sensitive populations to suppress the growth of the resistant population. The model was parameterized using values from Lu et al. (2024) (specified in Table S2).

#### Stem Cell Model

In contrast to the previous models, which categorize cells by their drug response, we also considered an alternate model proposed by Brady-Nicholls and Enderling (2022), which distinguishes between prostate cancer stem-like (*S*) and differentiated (*D*) cells to model the tumor response to treatment.

This model treats tumor growth as driven by a distinct stem cell population, rather than a drug-resistant sub-clone within the tumor bulk. These stem cells are immune to the drug and directly drive the growth of the drug-sensitive, differentiated population. As stem cells accumulate over the simulation, the growth rate of differentiated cells (that make up the bulk of the tumor) increases accordingly. Ultimately, the growth rate of differentiated cells (stimulated by stem cells) exceeds their drug-induced death rate, causing the cancer to progress during treatment. Importantly, the principles of AT still apply: progression is fundamentally driven, albeit indirectly, by expansion of the drug-resistant (stem cell) population, which can still be suppressed by a larger population of drug-sensitive (differentiated) cells.

Stem-like cells divide at a rate $\lambda$, to produce either two stem-like cells (with probability $p_{S}$, where $p_{S} < 1$, and subject to negative feedback $\frac{S}{S+D}$ from differentiated cells), or a stem-like and a non-stem cell. While stem-like cells are androgen-independent and hence do not respond to treatment, differentiated cells die in response to drug application at a rate $d_{D}$. A binary dosing schedule is implemented through $T_{x}$, where $T_{x}=1$ corresponds to treatment being given, and $T_{x}=0$ for treatment holidays.

3 $$\begin{aligned} \begin{gathered} \frac{dS}{dt} = \left( \frac{S}{S+D}\right) p_{S} \lambda S, \\\frac{dD}{dt} = \left( 1 - \frac{S}{S+D} p_{S}\right) \lambda S - d_{D} T_{x} D.\end{gathered} \end{aligned}$$

The model was parameterized using values from Brady-Nicholls and Enderling (2022) (specified in Table S2).

### Adaptive Therapy

We consider three distinct treatment protocols for the drug concentration *D*(*t*) (or $T_{x}(t)$ for the Stem Cell model) within this work. All drug doses are normalized such that $D(t)=1$ corresponds to the maximum tolerated dose.

1. **Continuous Therapy (CT)** – The standard of care:
  4 $$\begin{aligned} D(t) = 1\ \forall \ t. \end{aligned}$$
2. **Window-Based AT (AT50)** – Treatment is given until a decrease to $50\%$ of the initial size ($N_{0}$) is achieved, then withdrawn until the tumor returns to its initial size. AT50 was used in the pilot AT clinical trial by Zhang et al. (2017, 2022):
  5 $$\begin{aligned} D(t) = {\left\{ \begin{array}{ll} 1, \ until\ N(t)< 0.5 N_{0};\\ 0, \ until\ N(t) \ge N_{0}. \end{array}\right. }\end{aligned}$$
3. Threshold-based AT (AT-N*) – Treatment is given only when the tumor is larger than a set threshold size $N^{*}$:
  6 $$\begin{aligned} D(t) = {\left\{ \begin{array}{ll} 1, \ N(t) \ge N^{*};\\ 0, \ N(t) < N^{*}. \end{array}\right. } \end{aligned}$$

Treatment outcomes from these schedules are compared according to their TTP, where progression is defined as a 20 % growth from the initial size (i.e. $1.2 N_{0}$), as in prior studies in this area (e.g., Gallaher et al. (2018); Strobl et al. (2021); Viossat and Noble (2021)).

### Optimal Threshold

**Fig. 2:**
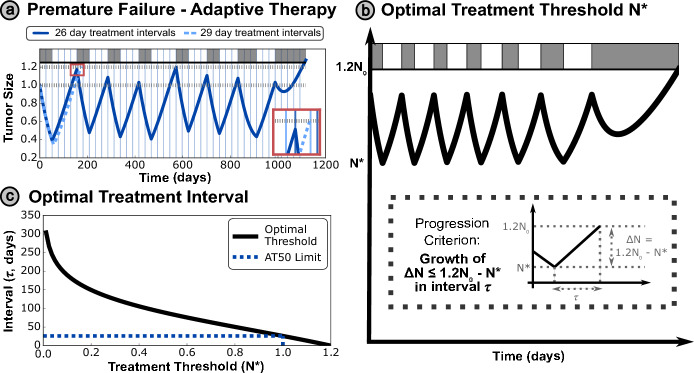
**(a)** Premature progression occurs when treatment decisions are made insufficiently frequently, such that the sensitive fraction of the tumor can grow from below the treatment threshold ($N(t) < 0.5 N_{0}$) to above the progression threshold ($N(t) > 1.2 N_{0}$ ) within a single treatment cycle. This places an upper limit on the maximum treatment threshold, given that patients are monitored at discrete intervals (denoted here by vertical lines). In this case, a small increase in the appointment interval (from 26 to 29 days) resulted in premature progression, shown in detail in the red inset (where the light blue, dashed simulation trajectory reaches progression just before the next appointment time, significantly decreasing the TTP). This figure uses a set of modified parameter values for visualization purposes, with $N_{0} = 0.3, d_{S} = 0.3 r_{S}$. **(b)** We can derive the optimal appointment interval $\tau ^{*}$ for a particular treatment threshold $N^{*}$ by ensuring that the time taken for the tumor to grow from the threshold to the progression limit is more than the time interval between appointments. **(c)** The required interval decreases monotonically as the treatment threshold is increased. The upper limit threshold of $N_{0}$ from conventional (‘window-based’) AT50 is indicated by the dashed line – this corresponds to an analytic limit on the AT appointment interval of 26.1 days, supporting the observation in (a)

We now consider the case of clinically realistic treatment protocols, limited by discrete time monitoring, and introduce the notion of an optimal threshold. The optimal threshold may be derived by considering a phenomenon we define as ‘premature progression’ - where insufficient treatment (or an overly long treatment holiday due to discrete time monitoring) results in progression due to growth in the sensitive cell population. The progression is deemed ‘premature’ as the tumor is still (partially) sensitive to treatment, and so progression could have been delayed by more frequent re-evaluation of treatment. Figure 2a demonstrates that even small increases in the interval between appointments may transform a successful strategy into one that undergoes progression in the first treatment cycle, with the tumor size *N*(*t*) jumping from $N(t) < 0.5 N_{0}$ (the threshold for treatment in conventional AT50) to $N(t) > 1.2 N_{0}$ (the threshold for progression) in a single treatment cycle.

We define the optimal appointment interval ($\tau ^{*}$ in Figure 2b) as the maximal possible time interval between treatment appointments, to avoid premature progression at a given treatment threshold. This interval may be obtained as the time for the tumor to grow from the threshold size for treatment to the progression limit. In practice, the interval between appointments is often determined by clinical availability and practical restrictions, so it is more practical to personalize the threshold tumor size $N^{*}$ within the AT protocol to each patient, based on a fixed time interval $\tau$.

We will apply this approach to each of the three tumor models introduced above, illustrating its applicability to different modeling frameworks. While the details of evaluating $N^{*}$ differ between models, and often require simplifying assumptions such as a negligible resistant population, the overarching approach is common between all models.

#### Lotka–Volterra Model

We assume $R=0$ such that the total tumor size *N* is equal to the sensitive population *S* (this assumption is justified in Supplementary Section S3.1). Rewriting (1), we obtain:

7 $$\begin{aligned} \frac{dN}{dt} = r_{S} N \left( 1 - \frac{N}{K}\right) - d_{S} N. \end{aligned}$$

Integrating over the optimal appointment interval $\tau ^{*}$, wherein the tumor grows from the threshold size $N^{*}$ to the progression limit $1.2N_{0}$:

$$\begin{aligned} \int _{N^{*}}^{1.2N_{0}}\frac{1}{r_{S} N \left( 1 - \frac{N}{K}\right) - d_{S} N} dN = \int _{0}^{\tau ^{*}} dt = \tau ^{*}, \end{aligned}$$

which may be evaluated to obtain:

8 $$\begin{aligned} \tau ^{*} = \frac{1}{r_{S}-d_{S}} \; ln \Biggl [ \frac{1.2N_{0}}{N^{*}} \frac{K(r_{S} - d_{S}) - r_{S} N^{*}}{K(r_{S} - d_{S}) - 1.2 r_{S} N_{0}} \Biggr ]. \end{aligned}$$

This relationship is plotted in Figure 2c - the appointment interval required decreases monotonically for larger treatment thresholds. Rearranging (8) to obtain an expression for the optimal treatment threshold $N^{*}$ based on a given appointment interval $\tau$, we obtain:

9 $$\begin{aligned} N^{*} = \frac{K(r_{S} - d_{S})}{\biggl (\frac{K(r_{S} - d_{S})}{1.2 N_{0}} - r_{S} \biggr )e^{(r_{S} - d_{S}) \tau } + r_{S}}. \end{aligned}$$

#### Waning Competition Model

Given that $r_{S} > r_{R}$, we again consider the growth rate of a wholly sensitive tumor (with negligible resistant fraction) and minimal inter-species competition ($\gamma = 0$), for the limiting case of fastest tumor recovery. In this case, the growth dynamics reduce to:

$$\begin{aligned} \frac{dS}{dt} = r_{S} S \left[ 1 - \left( \frac{S}{K_{S}}\right) ^{\alpha }\right] , \end{aligned}$$

which gives the integral (for the non-dimensionalized size $\hat{S} = \frac{S}{K_{S}}$):

$$\begin{aligned} \tau = \frac{1}{r_{S}} \int ^{\frac{1.2N_{0}}{K_{S}}}_{\frac{N^{*}}{K_{S}}} \frac{1}{\hat{S} \left( 1 - \hat{S}^{\alpha } \right) } d\hat{S}. \end{aligned}$$

Integrating, we obtain:

10 $$\begin{aligned} N^{*} = K_{S} \left[ \left( \left( \frac{K_{S}}{1.2N_{0}} \right) ^{\alpha } - 1 \right) e^{\alpha r_{S} \tau } + 1 \right] ^{-\frac{1}{\alpha }}. \end{aligned}$$

Note that this expression also holds for the widely-used generalized logistic model, as we assumed $\gamma = 0$ to consider the fastest possible growth for a tumor of given size but unknown composition.

#### Stem Cell Model

While in previous computations we have assumed that the drug-resistant population (i.e., the stem cell population *S*(*t*)) is negligible, that is not possible for this model, as the growth rate of the differentiated cells is directly proportional to the stem cell population.

We instead rewrite (3) during periods without treatment solely in terms of *N*, *S*:

$$\begin{aligned} \begin{gathered} \frac{dN}{dt} = \frac{dS}{dt} + \frac{dD}{dt} = \lambda S, \\ \frac{dS}{dt} = \left( \frac{S^{2}}{N}\right) p_{S} \lambda . \end{gathered} \end{aligned}$$

Hence, we have the separable equation:

11 $$\begin{aligned} \frac{dN}{dS} = \frac{N}{p_{S}S}, \end{aligned}$$

from which we derive an exact solution for the drug-free growth of the whole tumor. Considering the time interval $\tau$ taken for the tumor to grow from size $N^{*}$ to the progression limit 1.2*N*(0), we obtain the time-dependent expression for $N^{*}$:

12 $$\begin{aligned} N^{*}(t) = \left[ \left( 1.2N(0)\right) ^{\left( 1 -p_{S}\right) } - \frac{\lambda \left( 1-p_{S} \right) S(t)}{N^{*}(t)^{p_{S}}} \tau \right] ^{\frac{1}{1 -p_{S}}}, \end{aligned}$$

which we may solve numerically for $N^{*}(t)$. While $N^{*}$ depends on *S*(*t*), this can either be taken from ongoing measurements of the tumor, or evaluated by integrating (11) over the treatment history of the patient.

### Variable Offset Approach

If a treatment threshold $N^{*}$ larger than the optimal value for that tumor model is used, there is a risk of premature progression. However, this is not guaranteed, as our derivation for the optimal threshold considers a tumor that happens to have $N(t) = N^{*}$ at the start of the holiday period. If the tumor size is significantly smaller than the treatment threshold at the start of the holiday period, having overshot $N^{*}$ during the previous treatment period, then the tumor may not progress even when the threshold $N^{*}$ exceeds the optimal value. Our derivation, therefore, represents a ‘worst-case’ scenario to ensure that premature progression cannot occur.

To enable a direct comparison of the analytic derivation for the optimal $N^{*}$ with simulations of AT, we introduce an offset to the treatment schedule through an initial treatment period of duration $t < \tau$. This offset changes the time points at which treatment is subsequently re-evaluated, but does not change the interval $\tau$ between these time points, and so does not affect the optimal threshold. We then plot the minimum TTP from 20 simulations with different time offsets, replicating this ’worst-case’ scenario for that treatment protocol $(N^{*}, \tau )$. This approach reduces sensitivity to the timing of the first treatment, thereby avoiding the presentation of clinically unrealistic results. Further explanation of this approach is given in Supplementary Section S4.

## Results

### Lotka–Volterra Model

This paper aims to develop tools to improve patient outcomes by personalizing their treatment schedules. Before detailing how we propose to optimize scheduling, it is important to discuss the optimality criterion by which we seek to improve outcomes. Given the presence of a fully drug-resistant population in this model, it is not possible to eliminate the tumor entirely, which would cure the cancer. Furthermore, we show in Supplementary Section S2 that all the non-zero stationary states of the tumor are on the order of the carrying capacity *K*. We treat this size as an intolerable burden, where the tumor has taken over a host completely, and no more resources are available for growth. For this reason, in Section 2.2 we restrict the total tumor size $S(t) + R(t) = N(t) < 1.2 N_{0}$, where $N(t) = 1.2 N_{0}$ corresponds to clinical progression. Inevitably, the tumor will ultimately reach this progression limit, and hence, we also cannot contain the tumor at a tolerable size indefinitely. An optimal treatment strategy should therefore aim to delay tumor growth, thereby maximizing the time to reach the progression limit.

The net tumor growth rate is minimized when *N* is large, and so we adopt a threshold-based AT protocol, as further motivated in Supplementary Section S2.3. This protocol is based on the threshold $N^{*}$, which we derived in Section 2.3.1.

**Fig. 3:**
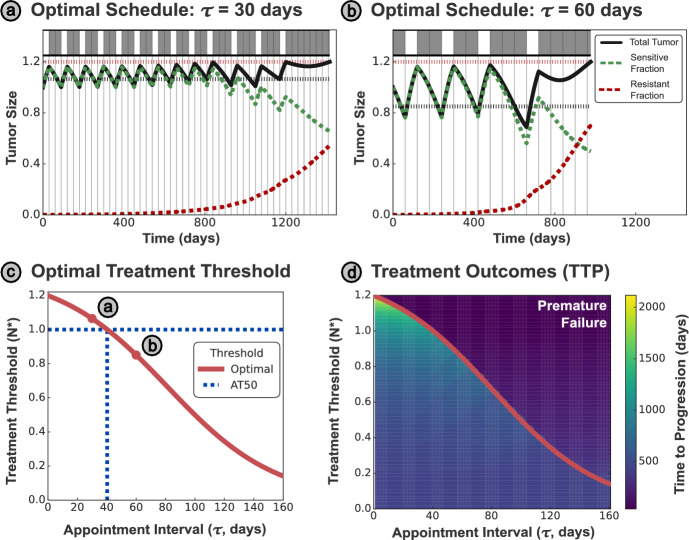
**(a-b)** Examples of AT-N* for $\tau = 30$ and $\tau = 60$ days, where the average tumor size is smaller under the 60 day interval, hastening tumor progression. For $\tau = 30$ days, the threshold $N^{*} > N_{0}$, and so the optimal strategy initially withholds treatment until the tumor exceeds the optimal threshold. **(c)** The analytically derived optimal threshold $N^{*}$ varies strongly with the appointment interval $\tau$, meaning that AT50 (where the upper threshold size is $N_{0}$) is only optimal for a specific appointment interval ($\sim 40$ days). Intervals shorter than this benefit from a higher threshold tumor size, while those longer require a smaller tumor size. **(d)** The curve $N^{*}(\tau )$ can be plotted over the space of simulated TTP outcomes, where each pixel corresponds to the TTP attained by an AT-N* protocol applied at an appointment interval $\tau$. The TTP is maximized at higher treatment thresholds; however, this is only possible with sufficiently frequent appointments. Treatment according to threshold values below $N^{*}$ (for a given appointment spacing) will be sub-optimal, while thresholds above $N^{*}$ have a risk of premature progression

Contrasting the optimal schedules for monthly and bimonthly appointments in Figure 3a-b, we can see that the higher treatment thresholds necessitate more frequent appointments. Plotting $N^{*}$ over $\tau$ in Figure 3c, we can see that this trend extends to the limit of continuous monitoring ($\tau = 0$), where the optimal threshold converges to $1.2N_{0}$ - the clinical threshold for progression. In contrast, an AT50 approach would only be possible if treatment is re-evaluated at least every 40 days, and longer intervals would risk premature progression. In the inset panels, we contrast the optimal treatment schedules for monthly (30 day) and bimonthly (60 day) appointment intervals. The shorter interval allows for better control over the tumor size, maintaining a more consistent and higher average size than the longer interval. The increased average tumor size results in greater suppression of the resistant cells, and hence greater overall TTP, but requires more frequent appointments to maintain. In other words, there is a trade-off between the cost and inconvenience of more frequent appointments against the higher TTP that a shorter appointment interval can attain.

We systematically evaluate the TTP for different combinations of threshold size and appointment interval in Figure 3b, reinforcing the argument that a higher threshold tumor size results in a greater TTP. However, this only applies when the treatment plan is re-evaluated sufficiently frequently (i.e., when the time interval between appointments is below the optimal treatment curve defined by (9) and plotted in red). Insufficiently frequent clinical appointments for a given threshold tumor size are inherently risky, resulting in poorer treatment outcomes (driven by premature progression) observed in the region above the optimal treatment curve.

### Waning Competition Model

**Fig. 4:**
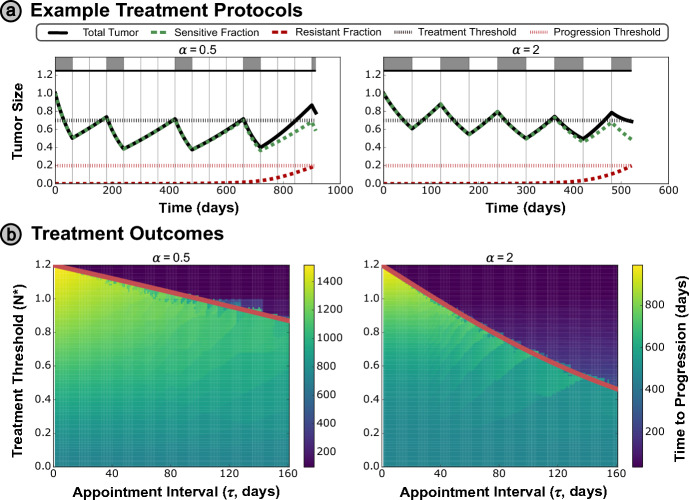
**(a)** Example treatment protocol (with $N^{*} = 0.7, \tau = 60$ days) for the Waning Competition model under two different values of the critical exponent $\alpha$ – note that progression is defined by the growth of the resistant population to $R(t) \ge 0.1 K_{R}$ for this model. **(b)** Treatment outcomes for differing $\alpha$ values, where the derived optimal threshold again accurately differentiates between premature progression above the optimal $N^{*}$ curve (red solid line), and the sub-optimal TTP outcomes below the curve

For the more complex Waning Competition model introduced in Section 2.1.2, we see that the exponent of the competition term $\alpha$ affects the dynamics significantly. We illustrate this through simulation of AT-N* treatment protocols (with $N^{*} =0.7, \tau = 60$ days) for two different values of $\alpha$ in Figure 4a.

The optimal threshold $$N^{*}$$, given in (10), is overlaid on the treatment outcome space for multiple values of $$\alpha $$ in Figure 4b, and separates the regions with and without the possibility of premature progression. While this more complex tumor model can account for a broader range of dynamics than the Lotka–Volterra model considered in Section 3.1, the TTP space depicts the same trends in the $$N^{*}$$ shown in the figure, and the optimal threshold framework outlined in Section 2.3 is equally applicable to this model. These results illustrate the translatability of the optimal threshold $$ N^*$$ and the framework we propose for deriving this threshold across different modeling assumptions and frameworks.

### Stem Cell Model

The mathematical models examined so far assume that the treatment dynamics are primarily driven by expansion and contraction of two tumor subpopulations with differential, but constant, fitness in the presence and absence of drug. One implication of this assumption is that, in the absence of logistic suppression, the single-species, per-capita growth rate ($\frac{1}{S}\frac{dS}{dt}$) remains constant over treatment. However, a subset of patients in the AT clinical trial by Zhang et al. (2022) (from which the data in Figure 1b were also taken) display qualitatively different tumor dynamics that cannot be replicated mathematically by the previous tumor models, most notably an increasing regrowth rate off-treatment after multiple treatment cycles.

**Fig. 5:**
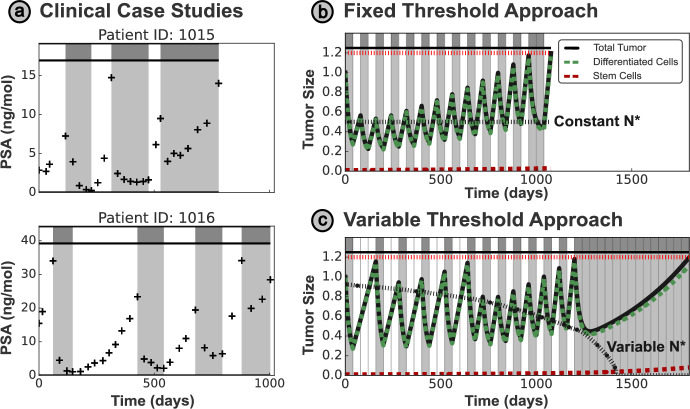
**(a)** Exemplar patient records from Zhang et al. (2022) demonstrate increasing rates of tumor rebound off-drug as treatment progresses. **(b)** This trend may be captured by the Stem Cell model of Brady-Nicholls and Enderling (2022); however, this behavior causes fixed threshold approaches to fail prematurely. In this plot, a fixed threshold of $N^{*} = 0.6$ for an interval $\tau = 40$ days is initially suboptimal, with the total tumor size less than half of the progression limit. Despite this, the fixed threshold protocol still allows premature progression (i.e., tumor progression off-treatment) by the end of the simulation. **(c)** For this model, a time-varying treatment threshold is required to ensure maximal suppression of the resistant population without resulting in premature progression - this approach significantly increases TTP over fixed threshold approaches with the same fixed appointment interval ($\tau = 40$ days).

Exemplified for two patients in Figure 5a, we see that subsequent holiday periods are shorter, as the tumor size recovers at an increasing rate on successive cycles of AT, in contrast to the approximately-constant holiday lengths for the patients in Figure 1b. One plausible explanation for such behavior is provided by the Stem Cell model proposed by Brady-Nicholls and Enderling (2022) (detailed in Section 2.1.2). Progression in this model is driven by an increasing population of stem cells, which eventually produce differentiated cells at a rate faster than they can be killed by the drug’s action.

Because the rate at which the tumor rebounds after treatment increases over time (as stem cells accumulate), a fixed threshold approach is no longer appropriate for this model. In Figure 5b, we see that the threshold $N^{*} = 0.5$ is suboptimal for a treatment interval of $\tau = 40$ days at the start of the simulation (maintaining a total tumor size significantly below the progression limit and hence weakening the competitive suppression of the drug-resistant stem cell population), while still failing to prevent premature progression occurring after approximately 1077 days. Applying our framework to derive $N^{*}$, we instead obtain a time-varying optimal threshold, as defined by (12).

While we cannot plot the TTP outcome over $(\tau , N^{*})$ space for time-varying $N^{*}(t)$, an exemplar treatment schedule for $\tau =40$ days is given in Figure 5c, which extends the TTP compared to the fixed threshold approach by 762 days. The threshold $N^{*}$ decreases over time to account for the faster tumor regrowth rate while the appointment interval is held constant, again exemplifying the trade-off between $N^{*}$ and $\tau$.

The concept of a time-varying treatment threshold is novel in AT and was not required in optimal treatment protocols for previous models, as they predict a constant rate of tumor regrowth over time. However, we have systematically shown that a time-varying threshold is optimal in specific modeling scenarios, such as the stem cell-driven tumor growth in this model, and is necessary to account for particular patient dynamics observed in previous clinical trials of AT. These dynamics are not unique to prostate cancer, and have also been observed in melanoma, where mathematical models with phenotypic plasticity (switching between drug-sensitive and -resistant states) have been proposed to account for this behavior (Kim et al. 2021). This heterogeneity in patient dynamics also raises wider questions regarding model selection for a given disease context, and whether multiple models should be used to capture qualitative heterogeneity in patient dynamics within a single study. Fundamentally, these results reflect the importance of tailoring mathematical models to the observed experimental/clinical behavior of the target system, enabling the translation of analytic results, such as optimal treatment protocols.

## Discussion

With multiple ongoing clinical trials of AT (in skin (NCT05651828 - BCC Trial), prostate (NCT05393791 - ANZADPT Trial), and ovarian (NCT05080556 - ACTOv Trial) cancers), it is of significant clinical interest to identify and characterize an optimal scheduling protocol for AT. However, previous work (Hansen et al. 2017; Viossat and Noble 2021) to identify optimal AT approaches does not account for discrete appointment intervals, which we show drastically modifies the ideal drug schedules.

We found that there is a trade-off between the appointment interval $\tau$ and the maximum attainable TTP, and introduced a threshold-based AT protocol that maximizes the TTP for a given appointment interval. This threshold $N^{*}$ depends on patient-specific tumor parameters, motivating the clinical need for personalized AT frameworks that can adapt to the dynamics of each patient. We also show that different mathematical models may be needed to capture qualitatively different patient dynamics, which can affect the optimal treatment strategy. To exemplify this, we identify patients with qualitatively different tumor dynamics (increases in the rate of tumor regrowth over time), and utilize a modeling framework that accounts for this behavior to propose a modified AT protocol where the treatment threshold may also vary over time. Our proposal that the best strategy may not just vary between patients, but also vary for a single patient throughout their treatment, is novel to AT. However, we hope this may inspire other innovative approaches to drug scheduling in different disease contexts.

While the cross-disease applicability of this framework extends beyond androgen suppression in prostate cancer, our approach relies on treatments that can be maintained outside clinical appointments. While patients traditionally had to attend the clinic to receive chemotherapy and other anti-cancer agents, it is increasingly common for these to be delivered at home, either orally (Jacobs et al. 2019; Moreira et al. 2022) or through drug infusion pumps (Sabbagh et al. 2021). This paper focuses on continuous treatments (such as daily pills); however, our framework could also be directly applied to protocols with discrete treatment cycles of a predetermined length, as is common for chemotherapeutic agents. As our generalized framework to derive $N^{*}$ only relies on the rate of tumor regrowth in the absence of treatment, this approach may be equally applied to treatment protocols with different treatment durations. However, our framework is only applicable when the elimination half-life of the relevant drug is significantly shorter than the holiday duration. This ensures that a binary dosing protocol with distinct max-dosage and drug-free periods may still be assumed. As the treatment period is now decoupled from appointment frequency, our framework would instead relate the treatment cycle length (the equivalent of $N^{*}$ for discrete treatment cycles) to the holiday duration directly. In contexts such as chemotherapy, where the treatment period is standardized, this would allow personalization of the holiday duration to maximize TTP.

To implement personalized AT protocols clinically, we also need an approach to estimate the individual’s tumor parameters before treatment. In previous work (Gallagher et al. 2024), we proposed a probing cycle to resolve this – all patients first undergo a standardized cycle of AT50, during which regular measurements of the tumor burden are taken. We may then fit our given mathematical model to these data to estimate the optimal $N^{*}$ threshold for that patient, and these fits may be iteratively refined as more data are collected from subsequent treatment cycles. This fitting process relies on our ability to derive a closed-form formula for $N^{*}$, and the parameter space may be simplified based on a sensitivity analysis of the $N^{*}$ formula. This emphasizes the importance of our analytic approach over a numerical estimation of $N^{*}$ from the original model equations.

Finally, we have discussed the role of modeling assumptions in this paper and how that may affect conclusions on the optimal treatment schedule. Our finding that different tumor dynamics necessitate different AT scheduling approaches motivates the development of a systematic approach to identify the most suitable tumor model for a specific patient’s dynamics, considering that these dynamics may vary qualitatively between tumors in the same disease context undergoing the same treatment. One approach to this could be an established suite of complementary mathematical models that may be fit to the same dataset, allowing systematic comparison of predictions between each model (Strobl et al. 2023) – such an approach may be implemented through an ‘Evolutionary Tumor Board’ that directly interfaces between mathematicians and clinicians to recommend optimal treatment protocols personalized to specific patients under the clinicians’ care (Robertson-Tessi et al. 2023). While our framework assumes perfectly periodic treatment intervals, we have separately proposed a framework to consider the sensitivity of such treatment schedules to appointment delays and predict the patient-specific risk of premature progression (Gallagher et al. 2015).

In summary, this paper illustrates the importance of accounting for clinical reality in the mathematical derivation of optimal treatment protocols, such as discrete patient monitoring. We show the importance of considering different underlying model assumptions based on the clinical data available, and demonstrate how these can lead to drastically different optimal treatment protocols, introducing a novel concept of an AT threshold that varies throughout a patient’s treatment. We hope this paper highlights the applicability of mathematical approaches to treatment scheduling in oncology and inspires future work to translate these analytic approaches into clinically actionable protocols across a range of disease settings.

## Supplementary Information

Below is the link to the electronic supplementary material.

Supplementary file 1 (pdf 593 KB)

## Data Availability

All code is available at https://github.com/KCGallagher/AT_Model_Comparison
