# Supplementary material for: Deriving Optimal Treatment Timing for Adaptive Therapy: Matching the Model to the Tumor Dynamics
Source: Bull Math Biol. 2025 Sep 8;87(10):146. doi: 10.1007/s11538-025-01525-y (PMC12417256; doi:10.1007/s11538-025-01525-y)
Supplement: Supplementary file 1 — (pdf 593 KB) [file 11538_2025_1525_MOESM1_ESM.pdf]

# Deriving Optimal Treatment Timing for Adaptive Therapy: Matching the Model to the Tumor Dynamics

Kit Gallagher, Maximilian A. R. Strobl, Alexander R. A. Anderson and Philip K. Maini

Oct 2024

## S1 Model Parameters

### S1.1 Lotka– Volterra Model

We adopt parameter values from Strobl et al. (2021), which the authors showed generate simulations that are consistent with the response dynamics observed in metastatic prostate cancer patients treated with hormone therapy. In cases where a range was given, we have considered the parameters that correspond to a 'worst case' scenario when the tumor is least responsive to treatment. As characterized in Strobl et al. (2021), this occurs when there is no cost to resistance (i.e. $r_S = r_R$) and no cell turnover (i.e. $d_S = d_R = 0$). The full list of parameter values used is given in Table S1.

| Name | Description | Value/Range | Reference |
|---|---|---|---|
| $r_S$ | Sensitive cell proliferation rate | $0.027\, day^{-1}$ | Adopted from Zhang et al. (2017) |
| $r_R$ | Resistant cell proliferation rate | $1.0 r_S$ | Zero cost scenario |
| $d_S, d_R$ | Natural cell death rate | 0 | Zero turnover scenario |
| $d_D$ | Drug-induced cell killing | 1.5 | Adopted from West et al. (2019) |
| $K$ | Tumor carrying capacity | 1 | Normalized to unity |
| $N_0$ | Initial tumor cell density | 0.75 | Rescaled by $K$ (Prokopiou et al., 2015) |
| $R_0$ | Initial resistant cell fraction | $0.001 N_0$ | Adopted from Grassberger et al. (2019) |

Table S1: Parameter values used for the Lotka– Volterra model, taken from Strobl et al. (2021).

### S1.2 Waning Competition Model

Parameter values for this model were taken from Lu et al. (2024), based on values that the authors obtained via a wide literature review, and are given in Table S2.

### S1.3 Stem Cell Model

Values for the model parameters were taken from Brady-Nicholls and Enderling (2022), which the authors obtained via fitting to clinical data. Parameter values from Patient 1014 were selected for visualization. However, values from Patients 1002, 1005, 1010, and 1018 were also used to verify that our approach is robust to variation in parameter values (results not shown, but the same qualitative trends were observed).

| Name | Description | Value |
|---|---|---|
| $r_S$ | Sensitive cell proliferation rate | $0.00715\, day^{-1}$ |
| $r_R$ | Resistant cell proliferation rate | $0.023\, day^{-1}$ |
| $K_S$ | Carrying capacity for sensitive cells | 1.0 |
| $K_R$ | Carrying capacity for resistant cells | 0.25 |
| $d_S$ | Drug-induced sensitive cell killing | 2 |
| $d_R$ | Drug-induced resistant cell killing | 0 |
| $S_0$ | Initial sensitive cell density | 0.5 |
| $R_0$ | Initial resistant cell density | $2.5 \times 10^{-5}$ |
| $\alpha$ | Growth scaling term | 1.0 |
| $\gamma$ | Relative competition | $0.0021\, day^{-1}$ |

Table S2: Parameter values used for the Waning Competition model, taken from Lu et al. (2024).

| Name | Description | Value |
|---|---|---|
| $\lambda$ | Stem-like cell proliferation rate | $0.69\, day^{-1}$ |
| $p_S$ | Symmetric division probability | 0.0425 |
| $\alpha$ | Drug-induced sensitive cell killing | $0.0478\, day^{-1}$ |
| $S_0$ | Initial stem-like cell population | 10 |
| $D_0$ | Initial differentiated cell population | 1000 |

Table S3: Parameter values used for the Stem Cell model, taken from Brady-Nicholls and Enderling (2022).

# S2 Motivation for AT under the Lotka –Volterra Model

In this section, we motivate the benefits of treatment breaks and AT protocols from a standard phase plane analysis of the Lotka –Volterra Model.

## S2.1 Stationary States

For notational simplicity, we let $\delta(t) = (1 - d_D D(t))$. While this may vary over time, we assume a constant value, denoted by $\delta$.

The non-trivial nullclines of this system (assuming non-zero values for each variable) are:

$$R = K\left(1 - \frac{d_S}{r_S\delta}\right) - S; \qquad R = K\left(1 - \frac{d_R}{r_R}\right) - S. \tag{S1}$$

Writing $\eta = \left(1 - \frac{d_S}{r_S\delta}\right)$ and $\mu = \left(1 - \frac{d_R}{r_R}\right)$, we obtain the nullclines:

$$R = \eta K - S; \qquad R = \mu K - S. \tag{S2}$$

From this, we may extract three stationary points:

$$(S, R) = (0, 0)\,, \quad (S, R) = (0, \mu K)\,, \quad (S, R) = (\eta K, 0)\,; \tag{S3}$$

in addition to the co-existence state $S + R = \eta K = \mu K$, in the special case where $\eta = \mu$.

We can think of $\eta$ and $\mu$ as the effective fitness of the sensitive and resistant populations, respectively. Negative values of $\eta$ or $\mu$ imply that the corresponding population is fundamentally unfit and would become extinct in isolation, in the absence of competition with the other species. In a biologically relevant parametrization, we would expect both cell populations to reach a steady state in a nutrient-limited monoculture, and hence both $\eta$ and $\mu$ should be positive. The only exception to this is where $\delta < 0$, i.e., the drug has a net-killing effect on the sensitive population, driving it to extinction even in the absence of competitive effects.

## S2.2 Stability Analysis

**Null State: $(S, R) = (0, 0)$** The eigenvalues of this state are given by:

$$\lambda = r_S\delta - d_S,\ r_R - d_R. \tag{S4}$$

Hence, this null state is only stable when $\eta$ and $\mu$ are both negative, a biologically implausible scenario, corresponding to a tumor that spontaneously becomes extinct even in the absence of treatment. Therefore, under biologically plausible parameterisations, complete tumor elimination is impossible, motivating the need to seek treatment schedules that instead reduce the net growth rate.

**Single-Species Exclusion: $(S, R) = (0, \mu K)\,;\ (\eta K, 0)$** The eigenvalues for the single-species exclusion state of cell type $i$ (excluding cell type $j$) are:

$$\lambda = d_i - r_i,\ r_j\delta\frac{d_i}{r_i} - d_j. \tag{S5}$$

While the first eigenvalue is always positive for biologically plausible parameterisations, the sign of the second depends on the relative fitness of the two states. By modulating $\delta$, the selective impact of the drug, we vary which single-species exclusion state is stable over treatment, enabling control over the relative proportion of sensitive and resistant cells.

### S2.3 Overall Dynamics

The nullclines of these ODEs are plotted in Figure S1a. We can formally consider the transition between the steady states as a bifurcation, where the number and nature of the stationary states change as $\eta - \mu$ changes sign. Varying these two parameters (representing the relative fitness of each population) relative to each other (plotted in Figure S1b) displays this change in stability of those states. Each species has a stable non-zero stationary state when its relative fitness is greater than that of the other species, while coexistence is possible when the species have the same relative fitness.

In all practical implementations of adaptive therapy, the progression limit $1.2N_0$ will be less than $min(\eta K, \mu K)$, and so the tumor will continue to grow to this progression limit for any treatment schedule. Adaptive therapy instead aims to drive the tumor over to the upper right region of phase space (Figure S1c) where the net tumor growth rate ($\frac{dN}{dt}$) on treatment is reduced, to delay progression for as long as possible.

The tumor growth is controlled via modulation of the drug term $\delta$: AT switches between the single-species exclusion states to keep $(1.2N_0 - N(t))$ small but strictly positive (i.e., a tumor that remains marginally smaller than the progression limit). To achieve this, we will establish an optimal tumor threshold. When the tumor exceeds this threshold size, the drug dose will be sufficient to ensure the overall size of a primarily sensitive tumor shrinks. Below the threshold size, the drug dose should be lowered (or removed entirely in the case of binary dosing protocols) to allow resensitization of the tumor.

## S3 Optimal Threshold Derivation

### S3.1 Non-Negligible Resistant Fractions

In Section 2.3.1, we have assumed that the resistant cell population ($R$) is negligible. While this may be true initially, this subpopulation will ultimately grow sufficiently to result in relapse. We therefore show that the critical appointment interval $\tau$, given by (8), still holds for any tumor composition $N = S + R$.

Let us suppose $r_S = r_R + \epsilon; d_R = d_S + \zeta$, where $\epsilon \geq 0; \zeta \geq 0$ - i.e. both the growth rate $r_S$ and the death rate $d_S$ of the sensitive cells are greater than or equal to the corresponding values for the resistant cells. This gives the following expression for the total tumor size $N$:

$$\frac{d}{dt}(S+R) = \left(1 - \frac{N}{K}\right)[r_S S + r_S R - \epsilon R] - d_S S - (d_S + \zeta) R. \tag{S6}$$

As before, we constrain this analysis to $N < K$, i.e., for tumor sizes less than the carrying capacity, because $N = K$ corresponds to non-positive growth rates of both the sensitive and resistant populations. We can therefore rewrite (S6) as:

$$\frac{d}{dt}(S+R) = r_S N\left(1 - \frac{N}{K}\right) - d_S N - R\underbrace{\left[\epsilon\left(1 - \frac{N}{K}\right) + \zeta\right]}_{+ve}, \tag{S7}$$

where the final term is non-negative. Contrasting to the growth rate of a fully sensitive tumor (7), we can see that the off-treatment growth rate of the mixed tumor is necessarily less than or equal to that of the fully sensitive tumor. This corresponds to a decrease in the critical appointment interval (8), since the mixed tumor will grow slower than the fully sensitive tumor, and so we will have more opportunities to intervene, and hence an increase in the maximum allowable treatment threshold (9). The critical threshold given by (9) is therefore exact when the resistant population is negligible (or there is no fitness difference between the two populations) and an overestimate otherwise. Given that the resistant fraction of the tumor will change over

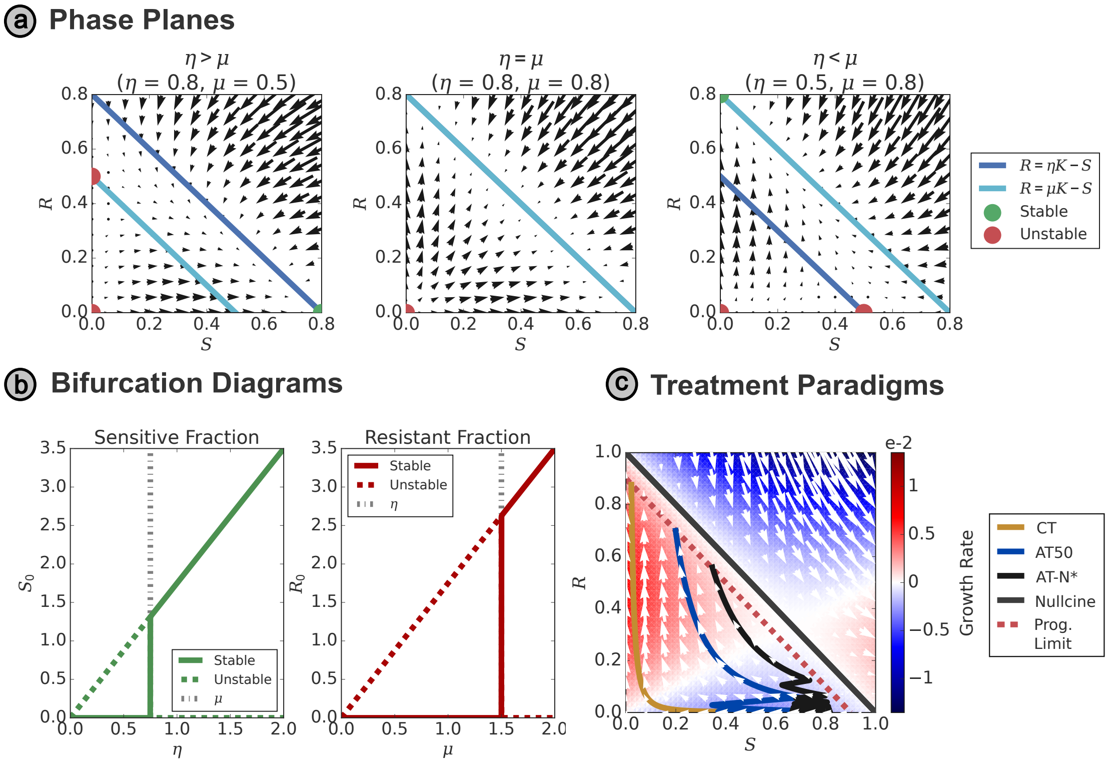


Figure S1: **(a)** Phase plane plots for different relative fitness of sensitive ($\eta$) and resistant ($\mu$) cells, with stationary states depicted by circles in each one. In the $\eta = \mu$ case, the two nullclines overlap, resulting in a continuum of stationary states. **(b)** Each species has a stable non-zero stationary state when its 'relative fitness' is greater than that of the other species. Coexistence is possible when the species have the same 'relative fitness' (where the stable state is a vertical line), while the other species has a stable non-zero stationary state (of constant size) below the bifurcation. **(c)** A comparison of different treatment protocols in phase space, superimposed over the net growth rate in the presence of the drug. AT maintains the tumor state close to the progression limit, where the net growth rate of the tumor is lower, resulting in delayed progression.

time, and is often not known at the start of treatment, we therefore conclude that (9) represents a 'worst-case' scenario.

# S4 Varied Offset Simulations

In Section 2.4, we discuss the possibility of premature progression (and significant reduction in TTP) based on the timing of treatment re-evaluation. To demonstrate this effect, we include an offset period (of duration $t < \tau$) at the start of the simulation - this merely changes the time points at which treatment is re-evaluated and does not affect the optimal threshold. For example, an offset of 20 days (when $\tau = 60$ days) means that treatment still starts at $t = 0$ but is subsequently re-evaluated at time points $t = 20, 80, 140...$ days. The regular threshold-based AT treatment protocol will be applied in the first treatment period, such that treatment will be given for the offset period if the initial tumor size $N(t = 0)$ is greater than the threshold size. Applying an offset period is equivalent to changing the initial size and composition of the tumor, while leaving the progression threshold and tumor dynamics unchanged.

An example of this is given in Figure S2a. Here we consider simulations of the Lotka–Volterra

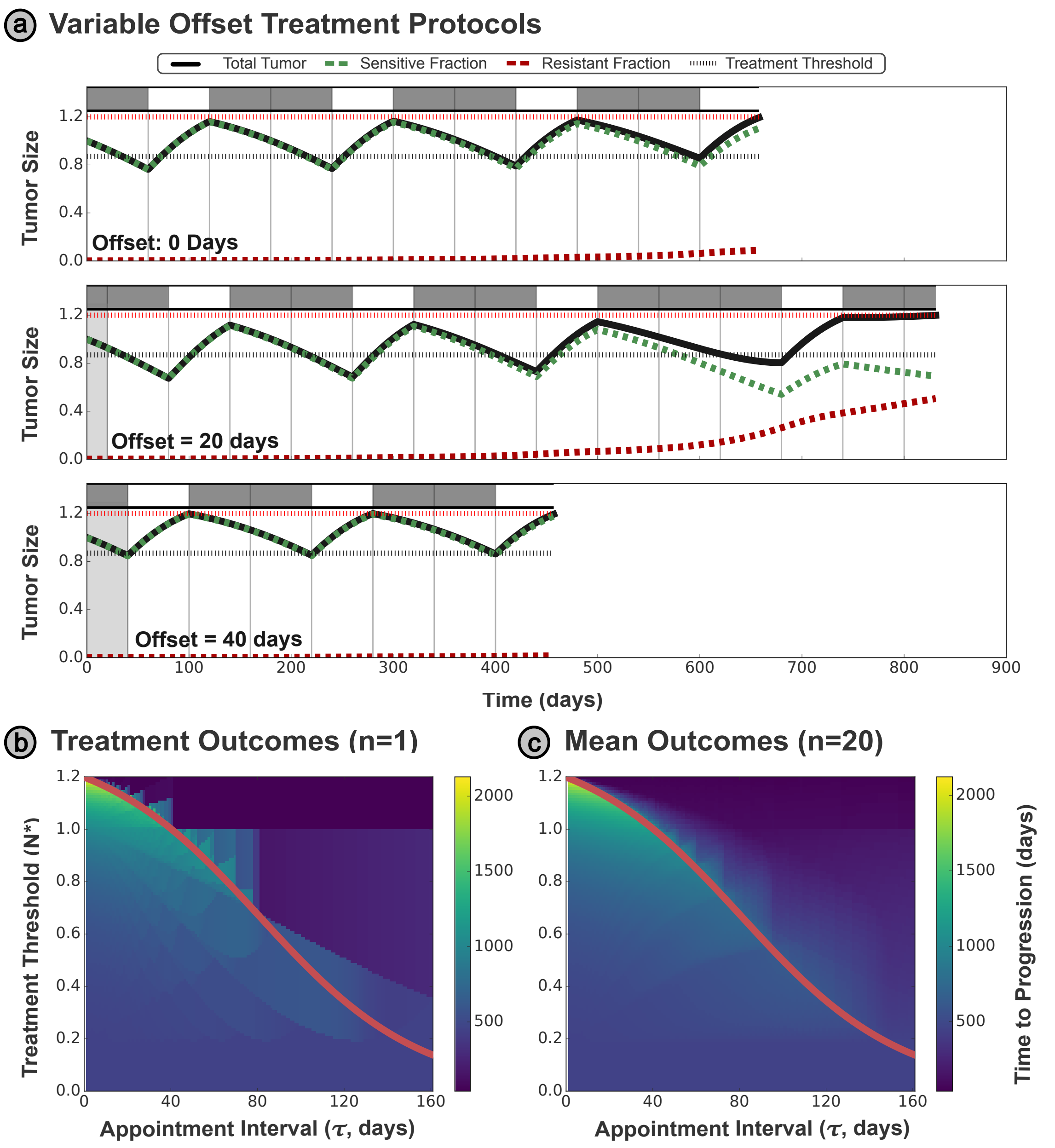


Figure S2: **(a)** Example treatment protocols (with a threshold size $N = 0.87$, $\tau = 60$ days) for the Lotka–Volterra model — this threshold is chosen to be slightly greater than the optimal value $N^* = 0.85$. Including varied time offsets in the appointment schedule demonstrates the sensitivity of the system and TTP to the precise timing of treatment. **(b)** Taking the TTP for a treatment schedule $(\tau, N^*)$ from a single simulation with no time offset results in highly variable TTP outcomes. This behavior is particularly accentuated just above the optimal threshold (given in red), where premature progression is possible but not guaranteed. **(c)** Computing the mean TTP from multiple simulations ($n = 20$) with different time offsets at the start of treatment allows us to mitigate the sensitivity to the treatment timing, with fewer simulation artifacts and discontinuities in TTP above the optimal threshold line.

model with an appointment interval of 60 days, where the corresponding optimal threshold is $N^* = 0.85$. To illustrate the risk of premature progression when a threshold greater than the optimal threshold $N^*$ is used, we simulate threshold-based AT with a slightly greater threshold

of $N = 0.87$. We see that the duration of the offset period drastically impacts the overall TTP - with an offset of 20 days the treatment schedule maintains control of the tumor until natural progression (driven by the resistant population), whereas differing offset values can result in premature progression at different time points, with an offset of 40 days approximately halving the maximal TTP. This extreme variation in TTP is also apparent in the full treatment outcomes space; when considering all combinations of $(\tau, N^*)$ in Figure S2b, we see that the occurrence of premature progression for thresholds greater than the optimal threshold is unpredictable. To mitigate this noise, we simulate each treatment schedule (defined by $\tau, N^*$) with multiple different offset values, and plot the mean TTP in Figure S2c. This figure displays a much smoother transition in TTP across the optimal threshold boundary.

Our derivation of the optimal threshold does not strictly predict the combinations of $(\tau, N^*)$ where premature progression definitely will occur; rather, it specifically allows us to differentiate between treatment schedules where it cannot occur and schedules where there is a possibility of premature progression occurring. This is intended to provide a minimal-risk treatment schedule, where there is no risk of premature progression even if clinical appointments are rescheduled, provided the maximal gap between appointments is not longer than $\tau$. For this reason, we present the treatment outcome spaces in the main text based on the minimum TTP obtained across a range of offset values, to replicate the 'worst-case' scenario in the clinic. In each case, we simulate the TTP subject to 20 different offset periods, of integer durations equally spaced between 0 and $\tau$, and plot the minimum TTP attained. If the appointment interval $\tau$ is less than 20 days, then we instead simulate for an offset given by each integer between 0 and $\tau - 1$ inclusive.

# References


Brady-Nicholls, R. and Enderling, H. (2022). Range-bounded adaptive therapy in metastatic prostate cancer. *Cancers*, 14(21):5319.

Grassberger, C., McClatchy, D., Geng, C., Kamran, S. C., Fintelmann, F., Maruvka, Y. E., Piotrowska, Z., Willers, H., Sequist, L. V., Hata, A. N., and Paganetti, H. (2019). Patient-specific tumor growth trajectories determine persistent and resistant cancer cell populations during treatment with targeted therapies. *Cancer Research*, 79(14):3776–3788.

Lu, Y., Chu, Q., Li, Z., Wang, M., Gatenby, R., and Zhang, Q. (2024). Deep reinforcement learning identifies personalized intermittent androgen deprivation therapy for prostate cancer. *Briefings in Bioinformatics*, 25(2).

Prokopiou, S., Moros, E. G., Poleszczuk, J., Caudell, J., Torres-Roca, J. F., Latifi, K., Lee, J. K., Myerson, R., Harrison, L. B., and Enderling, H. (2015). A proliferation saturation index to predict radiation response and personalize radiotherapy fractionation. *Radiation Oncology*, 10(1).

Strobl, M. A., West, J., Viossat, Y., Damaghi, M., Robertson-Tessi, M., Brown, J. S., Gatenby, R. A., Maini, P. K., and Anderson, A. R. (2021). Turnover modulates the need for a cost of resistance in adaptive therapy. *Cancer Research*, 81(4):1135–1147.

West, J. B., Dinh, M. N., Brown, J. S., Zhang, J., Anderson, A. R., and Gatenby, R. A. (2019). Multidrug cancer therapy in metastatic castrate-resistant prostate cancer: An evolution-based strategy. *Clinical Cancer Research*, 25(14):4413–4421.

Zhang, J., Cunningham, J. J., Brown, J. S., and Gatenby, R. A. (2017). Integrating evolutionary dynamics into treatment of metastatic castrate-resistant prostate cancer. *Nature Communications*, 8(1).
